# The Amyloid Precursor Protein C99 Fragment Modulates Voltage-Gated Potassium Channels

**DOI:** 10.33594/000000397

**Published:** 2021-07-28

**Authors:** Rían W. Manville, Geoffrey W. Abbott

**Affiliations:** Bioelectricity Laboratory, Department of Physiology and Biophysics, School of Medicine, University of California, Irvine, CA, USA

**Keywords:** Alzheimer’s disease, Potassium channel, KCNE, KCNQ2, KCNQ3

## Abstract

**Background/Aims:**

The Amyloid Precursor Protein (APP) is involved in the regulation of multiple cellular functions via protein-protein interactions and has been most studied with respect to Alzheimer’s disease (AD). Abnormal processing of the single transmembrane-spanning C99 fragment of APP contributes to the formation of amyloid plaques, which are causally related to AD. Pathological C99 accumulation is thought to associate with early cognitive defects in AD. Here, unexpectedly, sequence analysis revealed that C99 exhibits 24% sequence identity with the KCNE1 voltage-gated potassium (Kv) channel β subunit, comparable to the identity between KCNE1 and KCNE2–5 (21–30%). This suggested the possibility of C99 regulating Kv channels.

**Methods:**

We quantified the effects of C99 on Kv channel function, using electrophysiological analysis of subunits expressed in *Xenopus laevis* oocytes, biochemical and immunofluorescence techniques.

**Results:**

C99 isoform-selectively inhibited (by 30–80%) activity of a range of Kv channels. Among the KCNQ (Kv7) family, C99 isoform-selectively inhibited, shifted the voltage dependence and/or slowed activation of KCNQ2, KCNQ3, KCNQ2/3 and KCNQ5, with no effects on KCNQ1, KCNQ1-KCNE1 or KCNQ4. C99/APP co-localized with KCNQ2 and KCNQ3 in adult rat sciatic nerve nodes of Ranvier. Both C99 and full-length APP co-immunoprecipitated with KCNQ2 *in vitro*, yet unlike C99, APP only weakly affected KCNQ2/3 activity. Finally, C99 altered the effects on KCNQ2/3 function of inhibitors tetraethylammounium and XE991, but not openers retigabine and ICA27243.

**Conclusion:**

Our findings raise the possibility of C99 accumulation early in AD altering cellular excitability by modulating Kv channel activity.

## Introduction

The Amyloid Precursor Protein (APP) is involved in the regulation of multiple cellular functions via protein-protein interactions and has been most studied with respect to Alzheimer’s disease (AD). Abnormal processing of the C99 fragment of APP contributes to the formation of amyloid plaques, which are causally related to AD. In particular, increases in C99 levels in the brain are thought to contribute to cognitive defects in the early stages of AD. For example, increased C99 levels inside neurons were recently linked to defects in spatial information acquisition, alterations in synaptic plasticity, and early apathy-like behavior in the triple transgenic (3xTgAD: APPswe, Tau_P301L_, PS1_M146V_) mouse model of AD [1]. Toxic accumulation of C99 is also associated with early anatomical signatures of AD, and may even explain some undesirable side-effects of therapies that inhibit γ-secretase [2]. Importantly, C99 was previously identified as accumulating age-dependently and hippocampus-specifically in 3xTgAD mice, before Aβ accumulation, and with a timing consistent with a role in initiating the neurodegeneration and cognitive dysfunction in 3xTgAD mice [3].

Neuronal Kv channels, including the KCNQ channels that generate the widespread neuronal M-current (predominantly KCNQ2/3 heteromers), are important for learning and memory, and other cognitive functions. *De novo* KCNQ2 mutations cause early onset epileptic encephalopathies that include seizures but also severe cognitive impairment [4]. KCNQ2 is expressed more highly in human hippocampus than in other brain regions, and KCNQ2 gene variants and expression analysis suggest it is associated with cognitive decline in normal aging [5].

The KCNE family of K^+^ channel subunits (KCNE1–5) are single transmembrane (TM) domain glycoproteins that are formed from short polypeptides of ~130 amino acids. They co-assemble with a wide range of voltage-gated potassium (Kv) channel pore-forming α subunits, modifying channel functional properties including current density, gating kinetics, voltage dependence, trafficking and pharmacology. KCNE proteins are essential components of many Kv channels in a wide variety of organs and cell types throughout the body [6–8]. Consequently, inherited gene variants in *KCNE* genes are linked or associated with human diseases of abnormal electrical excitability, including Long QT syndrome, deafness, Brugada syndrome and periodic paralysis [9–13]. Further, *Kcne* gene deletion in mice causes a plethora of disorders ranging from cardiac arrhythmias [14] and deafness [15, 16] as in human *KCNE*-linked syndromes, to hypothyroidism [17], gastric cancer [18], atherosclerosis [19], diabetes [20] and increased seizure susceptibility [21].

Given that C99 and KCNEs each exhibit a single transmembrane span topology with an extracellular N-terminal domain and intracellular C-terminal domain, we examined their sequence similarity and the functional effects of C99 on a range of different Kv channel isoforms. Strikingly, C99 is highly effective at modulating Kv channel activity.

## Materials and Methods

### Channel subunit cRNA preparation and Xenopus laevis oocyte injection

We generated cRNA transcripts encoding human C99, KCNE1, KCNQ1, KCNQ2, KCNQ3, KCNQ4, KCNQ5, Kv1.1, Kv1.2, Kv4.2, Kv4.3S, Kv4.3L and hERG by *in vitro* transcription using the T7 polymerase mMessage mMachine kit (Thermo Fisher Scientific), after vector linearization, from cDNA sub-cloned into plasmids incorporating *Xenopus laevis* β-globin 5’ and 3’ UTRs flanking the coding region to enhance translation and cRNA stability. pCAX APP C99 cDNA was a gift from Dennis Selkoe & Tracy Young-Pearse (Addgene plasmid # 30146) [22]. We also generated cRNA from synthesized C99 cDNA (wild-type and with N- and C-terminal flag tag epitopes) and APP in pCDNA3.1+ (codon-optimized and with *Xenopus* β-globin 5’ and 3’ untranslated regions to enhance RNA stability and translation) (Biomatik, Wilmington, DE) to confirm results with a different source of plasmid and also facilitate biochemical analysis. We quantified cRNA by spectrophotometry. We injected commercially sourced, defolliculated stage V and VI *Xenopus laevis* oocytes (Ecocyte Bioscience, Austin, TX and Xenoocyte, Dexter, MI) with channel α subunit cRNAs (1–10 ng per oocyte depending on the isoform) with versus without C99 cRNA (5 ng per oocyte) or APP cRNA (7.5 ng/oocyte). We incubated the oocytes at 16 °C in Barth’s saline solution (Ecocyte) containing penicillin and streptomycin, with daily washing, for 2–5 days before two-electrode voltage-clamp (TEVC) recordings.

### Two-electrode voltage clamp (TEVC)

We conducted TEVC recordings at room temperature using an OC-725C amplifier (Warner Instruments, Hamden, CT) and pClamp8 software (Molecular Devices, Sunnyvale, CA) 2–5 days after cRNA injection, on oocytes placed in a small-volume oocyte bath (Warner) and viewed with a dissection microscope. We sourced chemicals from Sigma (St. Louis, MO, USA). The bath solution was (in mM): 96 NaCl, 4 KCl, 1 MgCl_2_, 1 CaCl_2,_ 10 HEPES (pH 7.6), and pipettes were of 1–2 MΩ resistance when filled with 3 M KCl. We recorded currents in response to pulses between −80 mV and + 40 mV at 20 mV intervals, or a single pulse to +40 mV, from a holding potential of −80 mV, to yield current-voltage relationships, current magnitude, and for quantifying activation rate. We quantified channel deactivation at −80 mV after a single pulse to +40 mV (of varying durations as indicated), from a holding potential of −80 mV. We performed electrophysiology data analysis using Clampfit (Molecular Devices), Origin (OriginLab Corporation, Northampton, MA, USA), and Graphpad Prism software (GraphPad, San Diego, CA, USA); values are stated as mean ± SEM. We plotted peak prepulse currents and raw or normalized tail currents against prepulse voltage and fitted the resultant plots with a single Boltzmann function:

Eq. 1: $$g=\frac{\left(A_{1}-A_{2}\right)}{\left\{1+exp\left[\left(V_{\frac{1}{2}}-V\right)/V_{\text{S}}\right]\right\}+A_{2}}$$

where *g* is the normalized tail conductance, A_1_ is the initial value at −∞, A_2_ is the final value at +∞, V_1/2_ is the half-maximal voltage of activation and V_s_ the slope factor. We fitted activation and deactivation kinetics with single exponential functions.

### Relative permeability calculations

According to the Goldman-Hodgkin-Katz (GHK) voltage equation:

Eq. 2: $$E_{rev\;}=\frac{RT/F\;ln\left(P_{K}{\left[K^{+}\right]}_{o}+P_{Na}{\left[Na\right]}_{o}+P_{Cl}{\left[Cl\right]}_{i}\right)}{\left(P_{K}{\left[K^{+}\right]}_{i}+P_{Na}{\left[Na\right]}_{i}+P_{Cl}{\left[Cl\right]}_{o}\right)}$$

Where *E*_rev_ is the absolute reversal potential and P is permeability. This permits calculation of the relative permeability of each ion if concentrations on either side of the membrane are known. We used a modified version of this equation to determine relative permeability of two ions in a system in which only the extracellular ion concentration was known. Thus, we calculated the relative permeability of Rb^+^, Cs^+^, and Na^+^ compared to K^+^ ions by plotting the I/V relationships with each extracellular ion (100 mM) and comparing them to the I/V relationship with 100 mM extracellular K^+^ ion, to yield a change in reversal potential (Δ*E*_rev_) for each ion compared to that of K^+^. We then calculated permeability ratios for each ion (X) compared to K^+^ as:

Eq. 3: $$\Delta E_{rev\;}=E_{rev{,}_{X}}-E_{rev{,}_{K}}=\frac{RT}{zF}\;ln\frac{P_{X}}{P_{K}}$$

### Chemical structures and silico docking

We plotted and viewed the retigabine chemical structure using Jmol, an open-source Java viewer for chemical structures in 3D: http://jmol.org/. For the *in silico* ligand docking prediction, we first altered the *Xenopus laevis* KCNQ1 cryoEM structure [23] to incorporate KCNQ3/KCNQ5 residues known to be important for retigabine and ML-213 binding, and their immediate neighbors, followed by energy minimization, as we previously described [24] using the GROMOS 43B1 force field [25] in DeepView [26]. We then performed unguided docking of retigabine using SwissDock with CHARMM forcefields [27, 28].

### Immunofluorescence

Frozen rat sciatic nerve sections were purchased from Zyagen (San Diego, CA), permeabilized (not fixed) in TBS with 0.1% Triton X-100, blocked for 1–2 h with 10% donkey serum, 1% BSA in TBS with 0.1% Triton X-100, then incubated overnight with primary antibodies diluted 1/100 in TBS with 0.1% Triton X-100: rabbit anti-APP (Thermo-Fisher), rabbit anti-C99 (Thermo-Fisher), rabbit anti-Ankyrin G (Santa Cruz Biotechnology), and goat anti-KCNQ2 or KCNQ3 (Santa Cruz Biotechnology). After washing 3 × 5 minutes, sections were incubated for 1–2 h in secondary antibodies (1/200 in TBS) raised in donkey (Invitrogen), before a final wash, mounted with DAPI-containing antifade solution, and then visualized on an Olympus BX51 microscope with Cell-Sens software (Olympus).

### Co-immunoprecipitations and western blotting

*Xenopus* oocytes expressing the various subunit combinations were frozen in batches following TEVC recording and stored at −20 °C until western blotting studies. Batches of 10 oocytes were lysed by suspension and repeated pipetting in a 1 ml pipette tip, in “MiRP buffer”: 150 mM NaCl, 50 mM Tris-HCL (pH 7.4), 20 mM NaF, 10 mM NaVO_4_, 1 mM phenylmethylsulfonyl fluoride (Thermo Fisher Scientific, Waltham, MA), 1% Nonidet P-40 (Thermo Fisher Scientific), 1% CHAPS (Sigma, St. Louis, MO, USA), 1% Triton X-100 (Thermo Fisher Scientific), and 1% SDS (Sigma) with protease inhibitor cocktail (Sigma). The suspension was rotated end-over-end at 4 °C for 2 hours, then centrifuged for 10 minutes at 5 × g at 4 °C. Supernatants were decanted (avoiding the pellet and uppermost layer) and used immediately for co-immunoprecipitations or frozen for lysate western blots.

For co-immunoprecipitation (co-IP), oocyte lysates were precleared with protein A agarose beads (ThermoFisher Scientific, Chino, CA, US) and then incubated overnight at 4 °C with one of the following antibodies: goat anti-KCNQ2 (Santa Cruz Biotech), rabbit anti-APP (Thermo-Fisher) or rabbit anti-FLAG (Thermo-Fisher). The following day, antibody-protein complexes were precipitated using protein A beads and separated on 4–12% Bis-Tris gels (Invitrogen), transferred onto PVDF membranes (Bio-Rad) and then western blotted as described below.

Oocyte lysates or co-IP beads were resuspended in LDS gel-loading buffer (Thermo Fisher Scientific) containing 25 mM tris(2-carboxyethyl)phosphine, heated for 10 minutes at 65 °C, vortexed, centrifuged for 3 minutes at 5 × g, and then the supernatants/bead eluents were separated by SDS-PAGE. Proteins were transferred (1 hour at 120 V) to PVDF membranes (BioRad, Hercules, CA, USA). After transfer, PVDF membranes were blocked in PBS (pH 7.6) containing 0.1% Tween-20 (PBST) and 5% dried milk for 1 h at room temperature, washed 3 × 5 minutes in PBST, and then incubated overnight at 4°C with antibodies as follows: 1/100 monoclonal mouse anti-KCNQ2 (Santa Crux Biotech), 1/200 rabbit anti-APP (Thermo-Fisher), 1/500 rabbit anti-FLAG (Thermo-Fisher) in PBST containing 5% (w/v) dried milk. Blots were next washed in PBST (3 × 5 minutes), incubated for 2 h at room temperature with 1/5000 Horseradish peroxidase (HRP)-conjugated IgG secondary antibodies (Bio-Rad or Thermo-Fisher) in PBST, and then washed again in PBST (3 × 5 minutes) before chemiluminescent detection using Luminata Forte chemiluminescence substrate (Millipore, Temecula, CA), and analysis using a Gbox system and Gbox software (Syngene).

### Statistical analysis

All values are expressed as mean ± SEM. One-way ANOVA was applied to determine statistical significance. All P-values were two-sided; statistical significance was defined as P < 0.05.

## Results

### Comparison of C99 and KCNE sequences

**Fig. 1.**
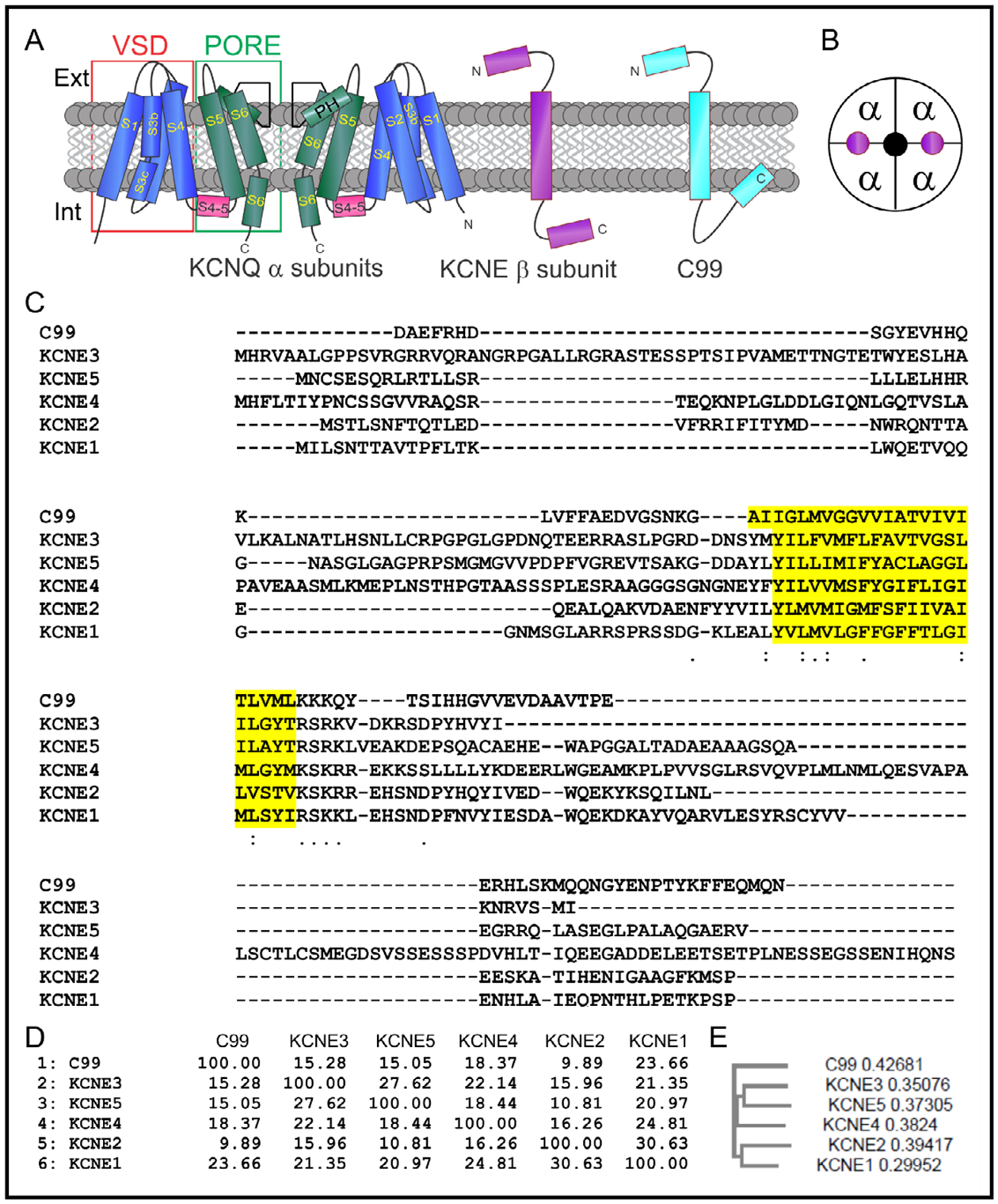
Sequence comparison of C99 and KCNE Kv channel β subunits. A: Topological representation of single KCNE and C99 subunits, and of a KCNQ channel showing two of the four subunits, without domain swapping for clarity. VSD, voltage sensing domain. Ext, extracellular; Int, intracellular. B: Cartoon of suggested 4:2 stoichiometry of a KCNQ1-KCNE1 Kv channel. Black circle, pore; magenta circle, KCNE subunit. C: Alignment of human C99 and KCNE protein sequences using EMBL-EBI MUSCLE. Yellow, predicted transmembrane region. D: Identity matrix of human C99 and KCNE protein sequences using Clustal2.1. Values are % sequence identities. E: Human C99/KCNE protein sequence phylogenetic tree generated in EMBL-EBI MUSCLE.

C99 and KCNEs share a common 1TM topology, each with extracellular N-terminal domains (Fig. 1A). Kv channel complexes, including those formed from KCNQs, contain 4 α subunits and may also contain 2–4 KCNE β subunits (Fig. 1A, B). C99 and KCNEs exhibit greatest sequence similarity in the transmembrane and intracellular membrane-juxtaposed regions (Fig. 1C). C99 similarity with KCNE1 is comparable to similarity among the KCNEs themselves (Fig. 1D), although the C99 sequence puts it outside the KCNE family (Fig. 1E).

### C99 isoform-specifically regulates KCNQ channel function

**Fig. 2.**
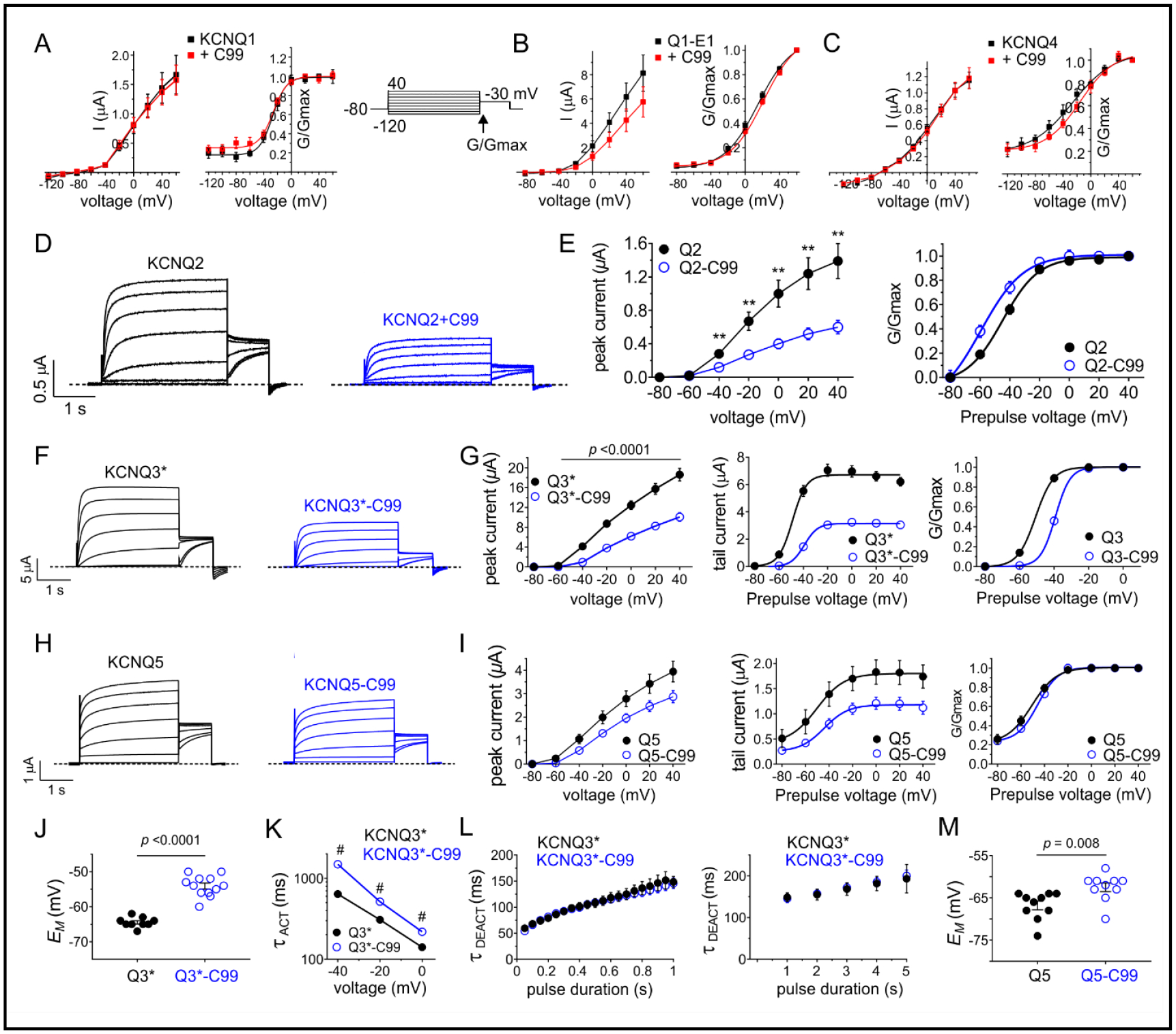
C99 isoform-selectively regulates KCNQ channel function. All error bars indicate SEM. Dashed lines indicate zero current level. A: *Left*, current/voltage (I/V) relationships; *right*, normalized tail current versus prepulse voltage (G/Gmax), for KCNQ1 alone (black; *n* = 11) or with C99 (red; *n* = 20) quantified by TEVC. *Right inset*, voltage protocol used for all similar data in this study. B: Data as in A for KCNQ1-KCNE1 (Q1-E1) alone or with C99; *n* = 9–10. C: Data as in A for KCNQ4 alone or with C99; *n* = 8–15. D: Averaged current traces for KCNQ2 alone or with C99; *n* = 20. E: *Left*, mean peak current versus voltage; *right*, G/Gmax, for KCNQ2 alone (black) or with C99 (blue); *n* = 20. **P = 0.002 for KCNQ2 versus KCNQ2-C99. F: Averaged current traces for KCNQ3* alone or with C99; *n* = 9–12. G: *Left*, mean peak current versus voltage; *center*, raw tail current versus prepulse voltage; *right*, G/Gmax, for KCNQ3* alone (black) or with C99 (blue); *n* = 9–12. H: Averaged current traces for KCNQ5 alone or with C99; *n* = 10. I: *Left*, mean peak current versus voltage; *center*, raw tail current versus prepulse voltage; *right*, G/Gmax, for KCNQ5 alone (black) or with C99 (blue); *n* = 10. J: Scatter plot showing effects of KCNQ3* alone or with C99 on unclamped oocyte membrane potential (E_M_); *n* = 9–12. K: Mean effects of C99 on KCNQ3* activation rate; *n* = 9–12. #P <0.0001. L: Lack of effects of C99 on KCNQ3* mean deactivation rate at −80 mV following different duration pulses to +40 mV; *n* = 10–12. #P >0.05. M: Scatter plot showing effects of KCNQ3* alone or with C99 on unclamped oocyte membrane potential (E_M_); *n* = 10.

We next tested the functional effects of C99 on KCNQ channels co-expressed in *Xenopus laevis* oocytes, using TEVC and a voltage family protocol (Fig. 2A upper inset). C99 had no effects on the cardiac and epithelial KCNQ1 channel, in the presence or absence of its cardiac and inner ear β subunit, KCNE1 (Fig. 2A, B). Neither did C99 alter KCNQ4 function (Fig. 2C).

In contrast, C99 exhibited the dual effect of reducing KCNQ2 peak current by >50% and negative-shifted its voltage dependence of activation by −10 mV (Fig. 2D, E). C99 also inhibited KCNQ3* current (a mutant form of KCNQ3 used to ensure robust currents through homomeric KCNQ3 channels [29]), but opposite to effects on KCNQ2, C99 positive-shifted KCNQ3* voltage dependence of activation, >10 mV (Fig. 2F, G). KCNQ5 was less sensitive to C99, which did not shift KCNQ5 voltage dependence of activation (Fig. 2H, I). KCNQ3*-C99 currents were still large enough to analyze in more detail without concerns about contamination from endogenous oocyte currents. Consistent with the positive shift on voltage dependence of KCNQ3* activation, C99 co-expression depolarized the resting membrane potential of oocytes expressing KCNQ3*, compared to those expressing KCNQ3* alone (Fig. 2J). Further, C99 slowed KCNQ3* activation but did not alter deactivation rate, suggesting that C99 destabilized the KCNQ3* open state (Fig. 2K, L). Finally, C99 also slightly depolarized the resting membrane potential of KCNQ5-expressing oocytes, consistent with its subtle effects on KCNQ5 function (Fig. 2M).

### C99 isoform-selectivity inhibits other Kv channels

**Fig. 3.**
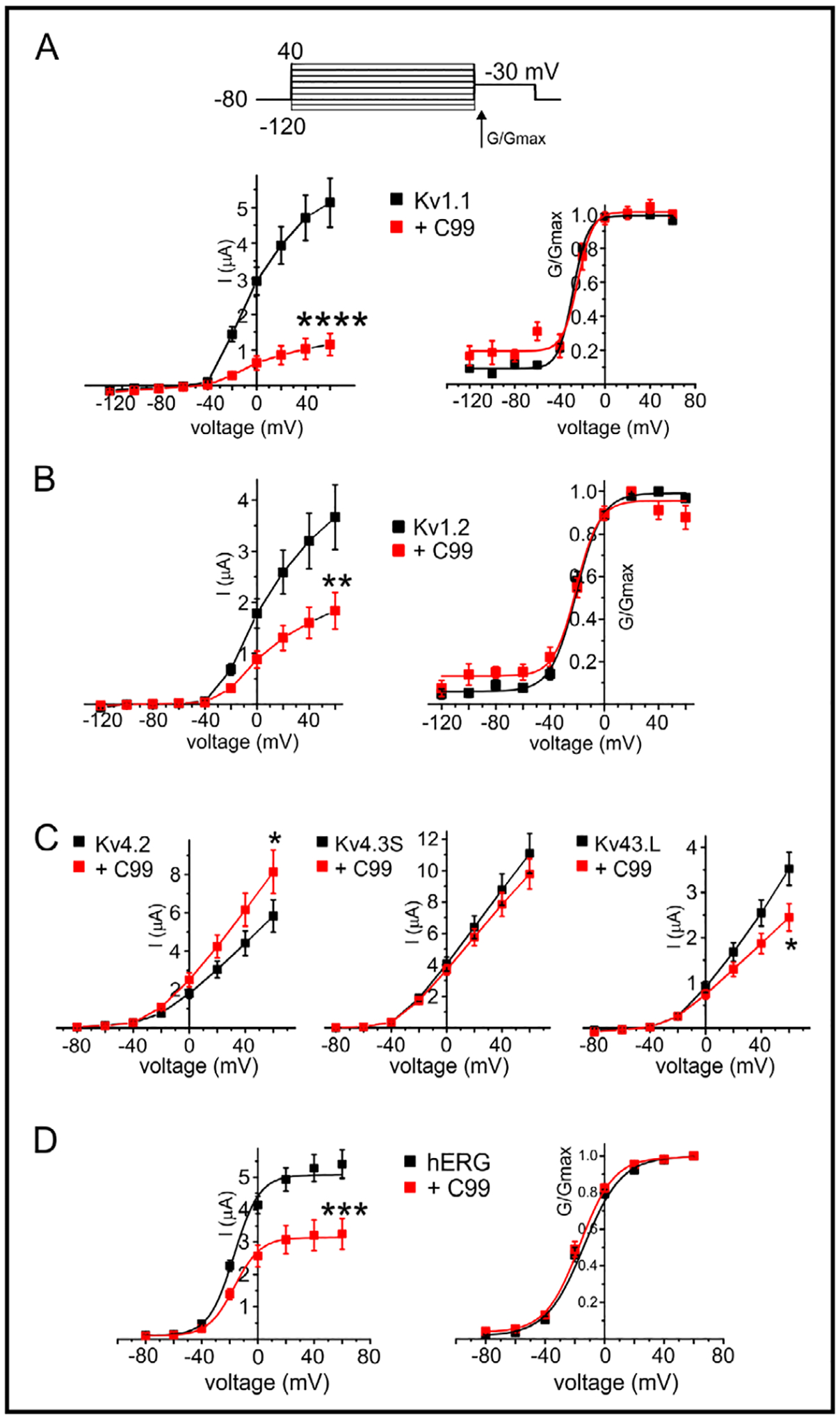
C99 has varied effects on other Kv channels. All error bars indicate SEM, all statistics at +40 mV. Mean peak current versus voltage relationships (*left*) and G/Gmax (*right; except panel C*) from traces recorded by TEVC in *Xenopus* oocytes expressing Kv channels alone (black) or with C99 (red); Voltage protocol, *upper center inset*. A: Kv1.1 with/without C99; *n* = 7–8; ****P = 3.4×10^−5.^ B: Kv1.2 with/without C99; *n* = 20–25; **P = 0.01. C: Kv4 channels as indicated with/without C99; *n* = 8–21; *P<0.05. D: hERG with/without C99; *n* = 22–23; ***P = 0.002.

C99 co-expression also inhibited other Kv channels, outside the KCNQ family. Thus, C99 diminished Kv1.1 (KCNA1) currents by 80%, and Kv1.2 by 50%, without altering the voltage dependence of either (Fig. 3A, B). Effects on Kv4 subfamily channels were varied and relatively small in magnitude: C99 slightly increased Kv4.2 current magnitude and decreased that of Kv4.3L, without changing Kv4.3S current magnitude (Fig. 3C). Finally, C99 decreased hERG current magnitude 40% without altering its voltage dependence (Fig. 3D).

### C99 regulates KCNQ2/3 channels

**Fig. 4.**
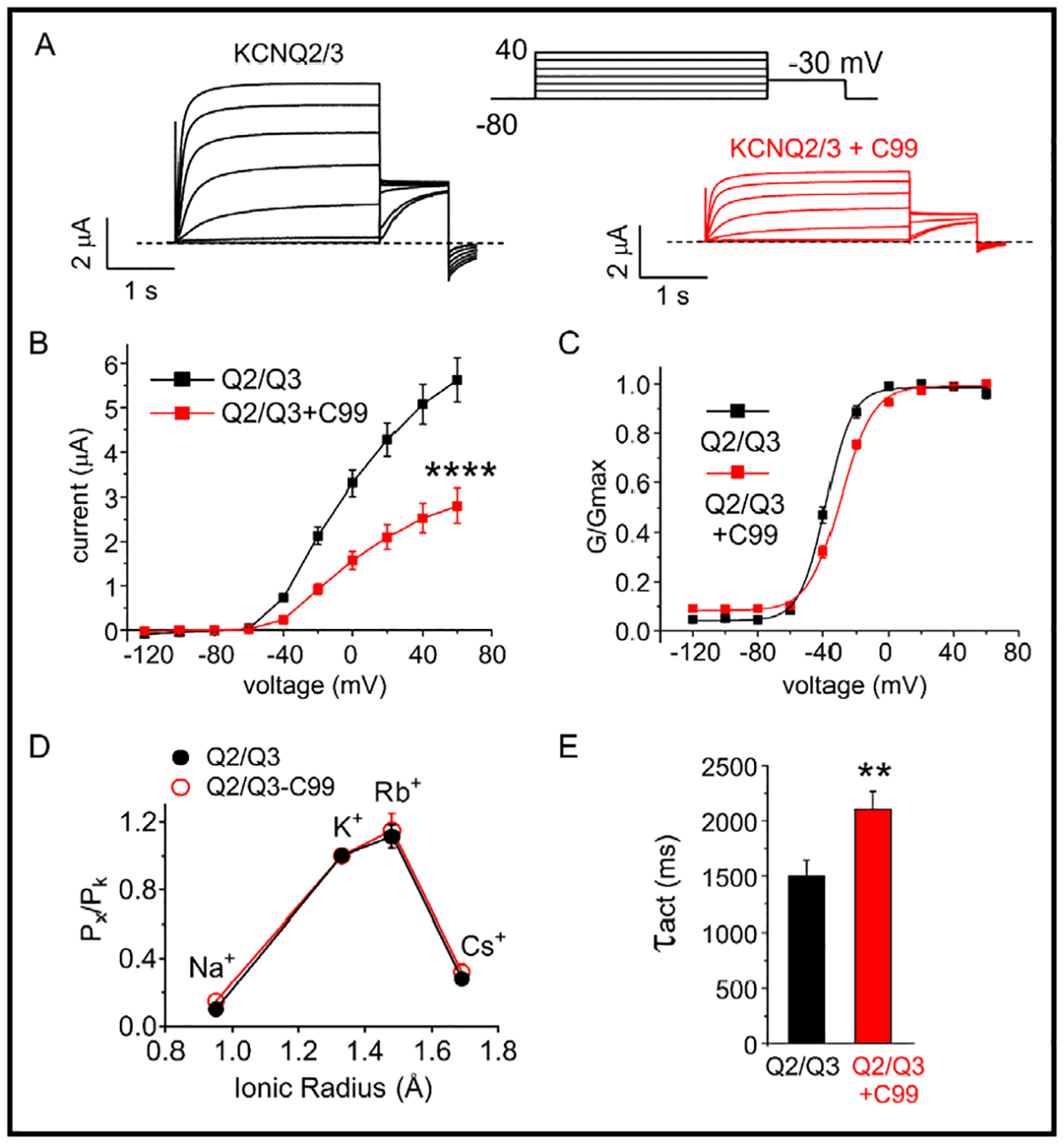
C99 regulates KCNQ2/3 channel function. All error bars indicate SEM. Dashed lines indicate zero current level. A: Mean current traces for KCNQ2/3 alone (black) or with C99 (red) quantified by TEVC using voltage protocol inset. B: Mean current/voltage (I/V) relationships for KCNQ2/3 alone (black; *n* = 25) or with C99 (red; *n* = 30) quantified by TEVC. ****P = 0.00003 at +40 mV. C: Mean normalized tail current versus prepulse voltage (G/Gmax), for KCNQ2/3 alone (black; *n* = 25) or with C99 (red; *n* = 30) quantified by TEVC. D: Lack of effects of C99 on KCNQ2/3 ion selectivity quantified by measuring various ion permeabilities relative to that of K^+^; *n* = 10–12. E: Mean activation rate for KCNQ2/3 alone or with C99 as indicated; *n* = 25–30. **P = 0.006.

Heteromeric KCNQ2/3 channels are considered to be the predominant molecular correlate of the muscarinic-inhibited neuronal M-current, which is important for controlling neuronal excitability. C99 inhibited KCNQ2/3 peak current by 70%, positive-shifted its voltage dependence of activation and also changed the slope of KCNQ2/3 voltage dependence (Fig. 4A–C). C99 did not alter the ion selectivity of KCNQ2/3 channels (Fig. 4D) but slowed their activation (Fig. 4E).

### C99 and APP co-localize with KCNQ2/3 in rat sciatic nerve and form complexes in vitro

**Fig. 5.**
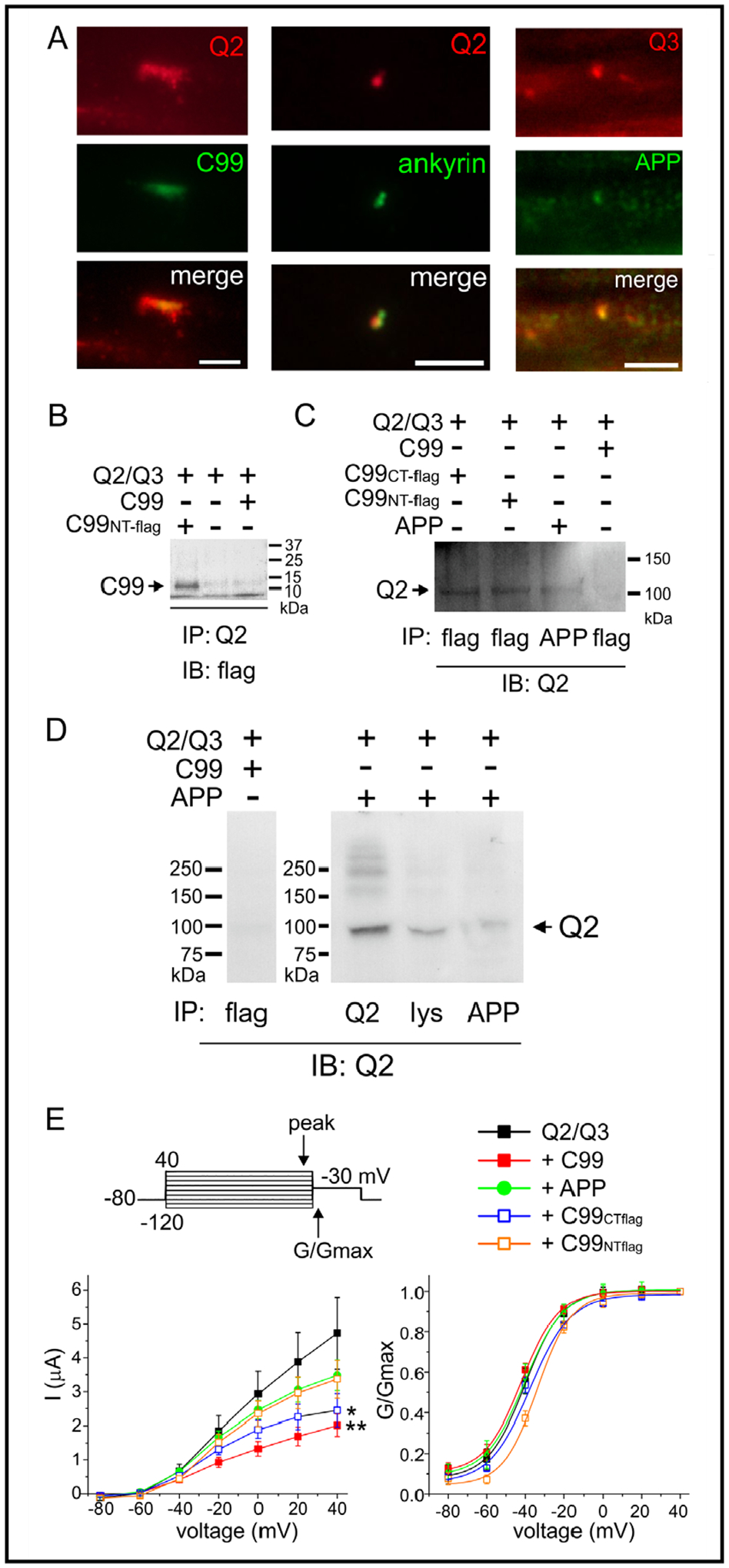
C99/APP co-localizes with KCNQ2/3 in rat sciatic nerves and co-assembles with KCNQ2/3 *in vitro*. A: Representative (from at least two different days, and >2 sections per day, for each combination) fluorescence images of adult rat frozen sciatic nerve nodes of Ranvier double-immunostained using antibodies and colors as indicated. Merge indicates merged image of two color channels. Scale bars = 2 μm. Left, KCNQ2 and C99; center, KCNQ2 and Ankyrin G (nodal marker); right, KCNQ3 and APP. B: Western blot showing co-immunopreciptation of N-terminal flag-tagged C99 (C99NT-flag) with KCNQ2 (Q2), subunit expression indicated above with + symbols. IP, immunoprecipitation; IB, immunoblot. C: Western blot showing co-immunopreciptation of KCNQ2 (Q2) with N- and C-terminal flag-tagged C99 and with APP, but not untagged C99 (when co-IPing with anti-flag antibody). Subunit expression indicated above with + symbols. IP, immunoprecipitation; IB, immunoblot. D: Western blot showing co-immunopreciptation of KCNQ2 (Q2) with APP (using anti-APP antibody), but not untagged C99 (when co-IPing with anti-flag antibody); as a positive control KCNQ2 was IPed with anti-KCNQ2 antibody. Subunit expression indicated above with + symbols. IP, immunoprecipitation; IB, immunoblot; lys, lysate control. E: *Left*, mean peak current/voltage (I/V); *right*, mean G/Gmax relationships, for KCNQ2/3 alone (black; *n* = 12) or with synthetic cDNA construct derived subunits as follows: wild-type C99 (red; *n* = 16), C-terminal flag-tagged C99 (red; *n* = 13), N-terminal-tagged C99 (red; *n* = 11), wild-type APP (red; *n* = 17) quantified by TEVC. *P = 0.05; **P = 0.01 versus KCNQ2/3 alone at +40 mV; others P>0.05. Voltage protocol, upper left inset.

We and others previously showed that KCNQ2/3 channels are found, among other locations, at the nodes of Ranvier in rat sciatic nerve [30, 31]. Interestingly, others found that APP, the protein that is cleaved to produce C99, also localizes to nodes of Ranvier [32]. Accordingly, here we found that both KCNQ2 and KCNQ3 co-localize in rat sciatic nerve nodes of Ranvier with proteins detected using antibodies raised against C99 and/or APP (Fig. 5A). This suggests that C99 and/or APP regulation of KCNQ2/3 could occur *in vivo*.

We next generated FLAG-tagged C99 (N- and C-terminal tagged versions) and also wild-type C99 and APP, using synthetic cDNA constructs, to facilitate analysis of complex formation in oocytes. Flag-tagged C99 and wild-type APP each co-immunoprecipitated with KCNQ2 (Fig. 5B–D). As negative controls, untagged C99 was not detected with anti-FLAG antibody (Fig. 5B) and KCNQ2 was not immunoprecipitated with untagged C99 when using an anti-FLAG antibody to immunoprecipitate (Fig. 5C, D).

We also compared the effects of the synthetic construct-generated C99 and APP on KCNQ2/3 function. Synthetic wild-type C99 inhibited KCNQ2/3 by >50%, similar to findings for the original wild-type C99 construct. The FLAG tags partially impaired C99 ability to inhibit KCNQ2/3, possibly indicating effects of the flag tag on either expression or functionality. APP only weakly inhibited KCNQ2/3 function, suggesting cleavage to C99 would increase inhibition within APP-KCNQ2/3 complexes. Effects of any of the proteins on KCNQ2/3 voltage dependence were marginal, as observed for the original wild-type C99 construct (Fig. 5E).

### C99 modifies the pharmacological profile of KCNQ2/3 channels

**Fig. 6.**
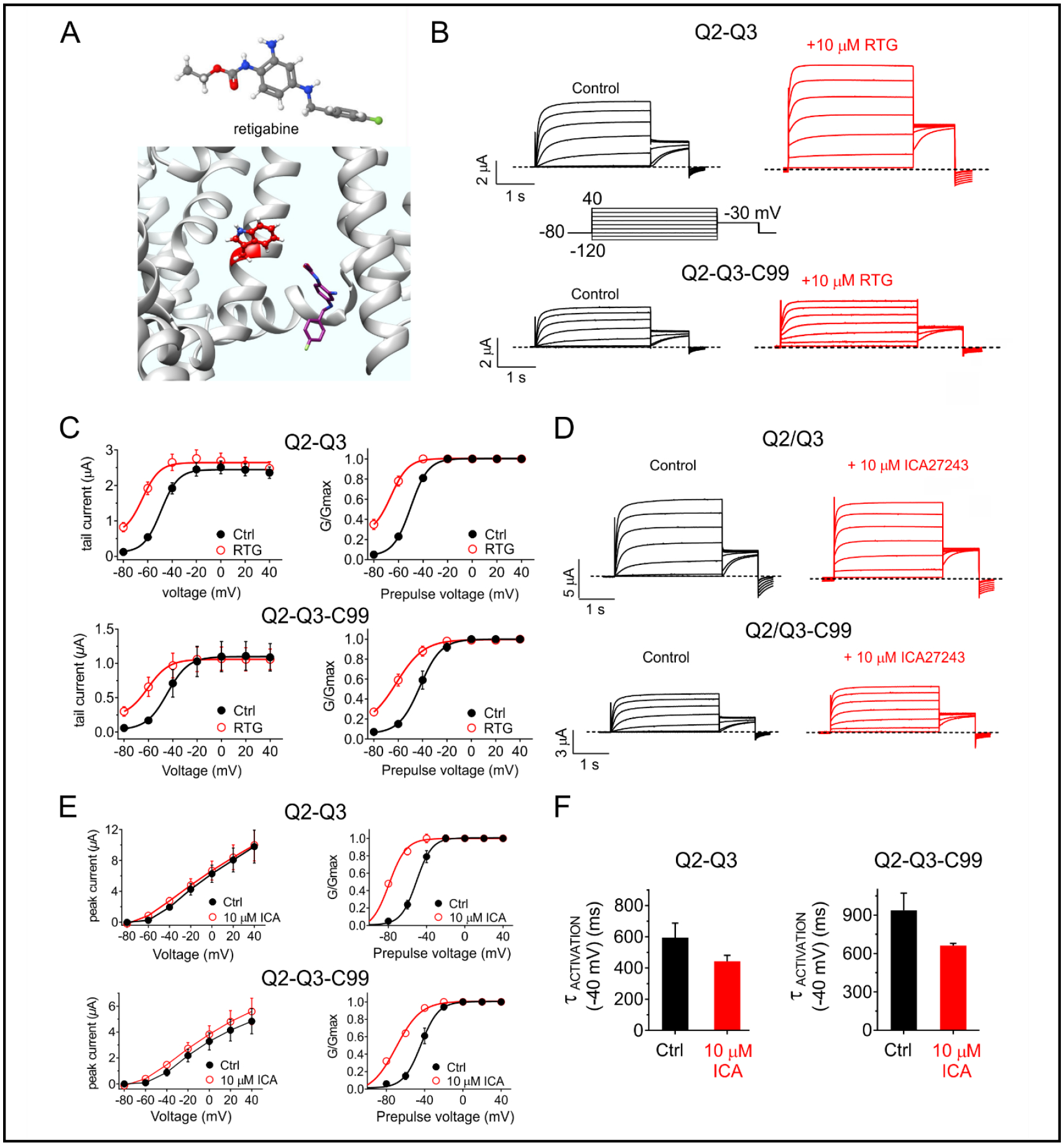
C99 does not prevent KCNQ2/3 current potentiation by pharmacological openers. All error bars indicate SEM. Dashed lines indicate zero current level. A: *Upper*, retigabine structure; *lower*, *in silico* docking of retigabine close to KCNQ3-W265. B: Mean TEVC traces for KCNQ2/3 with or without C99 in the absence (Control) or presence of 10 μM retigabine (RTG) (*n* = 6–10). Voltage protocol, *center inset*. C: Mean tail current (*left*) and normalized tail currents (G/Gmax; *right*) versus prepulse voltage relationships recorded by TEVC in *Xenopus* oocytes expressing KCNQ2/3 alone or with C99 in the absence (black) or presence (red) of 10 μM RTG as indicated (*n* = 6–10). D: Mean TEVC traces for KCNQ2/3 with or without C99 in the absence (Control) or presence of 10 μM ICA27243 (*n* = 5–8). Voltage protocol as in B. E: Mean peak current (*left*) and normalized tail currents (G/Gmax; *right*) versus prepulse voltage relationships recorded by TEVC in *Xenopus* oocytes expressing KCNQ2/3 alone or with C99 in the absence (black) or presence (red) of 10 μM ICA27243 as indicated (*n* = 5–8). F: Mean effects of 10 μM ICA27243 on the activation rate of KCNQ2/3 alone or with C99 in the absence (black) or presence (red) of 10 μM ICA27243 as indicated (*n* = 5–8).

KCNQ2/3 is regulated by a number of small molecules, and new compounds are being specifically developed to open KCNQ2/3 channels with the aim of preventing or treating epilepsy. Retigabine is a KCNQ2/3 opener that requires the S5 residue KCNQ2-W236/KCNQ3-W265 for channel binding and/or activation [33–35] (Fig. 6A). Here, we found that retigabine did not rescue KCNQ2/3 activity from the inhibitory effects of C99. Rather, retigabine exerted similar fold-effects on KCNQ2/3 and KCNQ2/3-C99 channels such that in the presence of drug, C99 was still able to influence KCNQ2/3 activity (Fig. 6B, C). We observed a similar pattern with ICA27243, a KCNQ2/3 opener that is thought to bind to the KCNQ2/3 voltage sensors [36] (Fig. 6D–G).

**Fig. 7.**
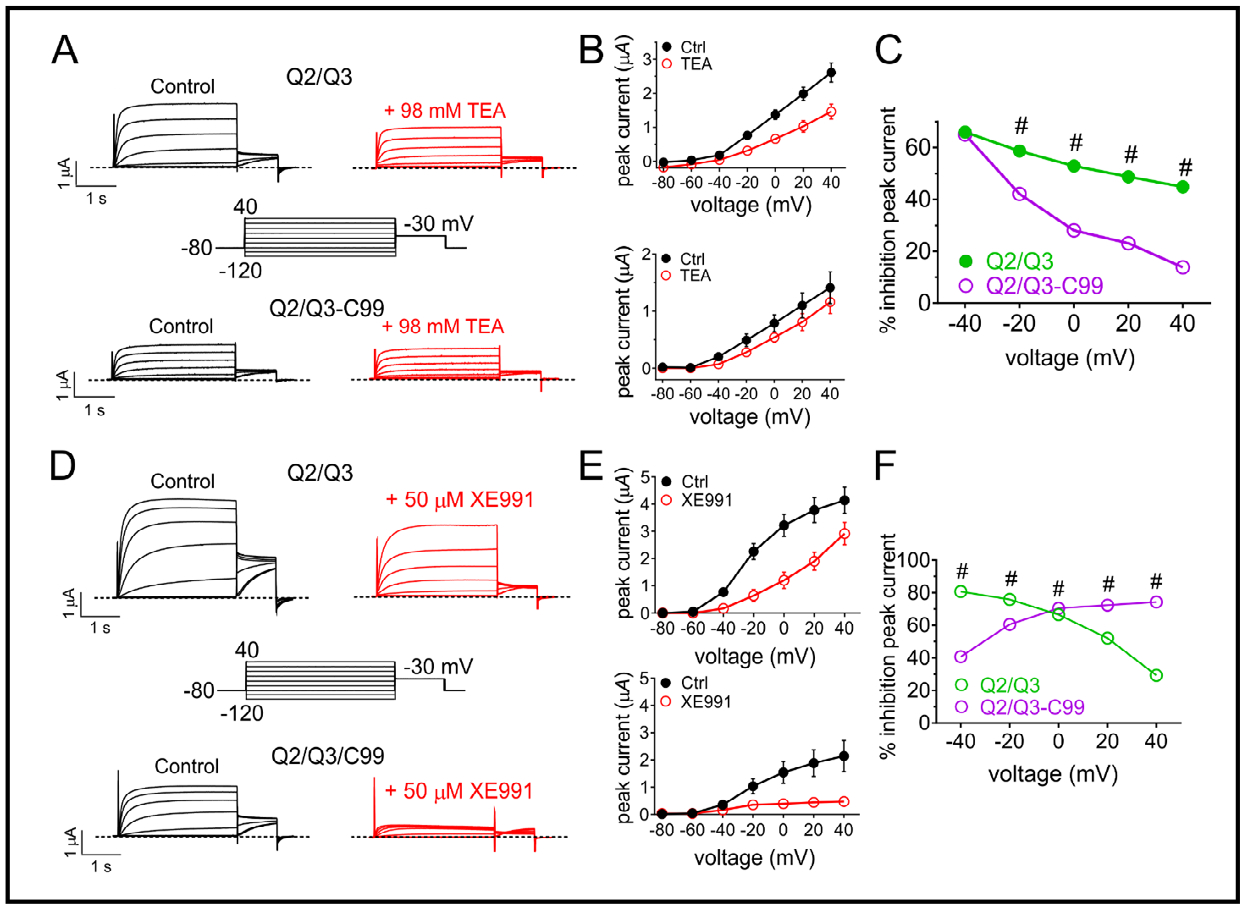
C99 alters the effects of inhibitors on KCNQ2/3. All error bars indicate SEM. Dashed lines indicate zero current level. A: Mean TEVC traces for KCNQ2/3 with or without C99 in the absence (Control) or presence of 98 mM TEA (*n* = 6–8). Voltage protocol, *center inset*. B: Mean peak current versus voltage relationships recorded by TEVC in *Xenopus* oocytes expressing KCNQ2/3 alone or with C99 in the absence (black) or presence (red) of 98 mM TEA (*n* = 6–8) for traces as in A. C: Mean peak current inhibition by TEA of KCNQ2/3 alone (green) or with C99 (purple) for traces as in A; *n* = 6–8; # P <0.0001. D: Mean TEVC traces for KCNQ2/3 with or without C99 in the absence (Control) or presence of 50 μM XE991 (*n* = 8–10). Voltage protocol, center inset. E: Mean peak current versus voltage relationships recorded by TEVC in Xenopus oocytes expressing KCNQ2/3 alone or with C99 in the absence (black) or presence (red) of 50 μM XE991 (*n* = 8–10) for traces as in D. F: Mean peak current inhibition versus voltage by 50 μM XE991 of KCNQ2/3 alone (green) or with C99 (purple) for traces as in D; *n* = 8–10; # P <0.0001.

Tetraethylammonium (TEA) is a potassium channel pore blocker that voltage-dependently inhibits many different Kv channels with varying efficacies and potencies [37, 38]. KCNQ2/3 channels are relatively insensitive to TEA but can be blocked by high millimolar amounts. Here, C99 reduced the efficacy of 98 mM TEA on KCNQ2/3, with the most pronounced effects at higher positive voltages (e.g., a threefold reduction in efficacy at +40 mV) (Fig. 7A–C). XE991 is a relatively KCNQ-specific, state-dependent inhibitor thought to bind to a single activated KCNQ2 subunit rather than being an open-channel inhibitor [39]. C99 reversed the voltage dependence of KCNQ2/3 inhibition by XE991, decreasing efficacy at negative potentials but increasing efficacy at positive potentials (e.g., by >twofold at +40 mV (Fig. 7D–F).

## Discussion

APP was previously found to be expressed at nodes of Ranvier, a prime location for potentially regulating ion channel activity given that there are both Kv channels and voltage-gated sodium channels located there. Here, we noted that the C99 fragment of APP bears topological similarity and some sequence similarity to the KCNEs, a well-known family of 1TM Kv channel β subunits. Further, we found that C99 is capable of regulating a variety of Kv α subunits. While the predominant effect of C99 was to inhibit Kv channel activity, this was not the only feature of its effects. Thus, C99 modulated the voltage dependence of KCNQ2, KCNQ3, and KCNQ2/3 channel activation and also the activation rate of these channels.

These data are consistent with C99 forming complexes with KCNQ2/3 in the plasma membrane and directly regulating its activity there, as supported by co-immunoprecipitation results (Fig. 5). Also consistent with this, and not explicable by indirect regulation, C99 altered the pharmacology of KCNQ2/3. Specifically, C99 reduced the efficacy of TEA, diminishing its inhibition of KCNQ2/3, particularly at more positive membrane potentials. Conversely, C99 augmented the inhibitory effects of XE991 at positive membrane potentials. TEA is a pore blocker that does not cross the membrane [37, 38]. It is possible that the extracellular domain of C99 protects the outer pore TEA binding site in KCNQ2/3, analogous to the protective effect of specific residues (and a glycosylation site) in the KCNE2 extracellular domain in protecting hERG from drug block [10, 40]. XE991, in contrast, inhibits KCNQ2/3 by binding to single activated subunits within the tetramer, and is not thought to be a pore blocker [39]. The augmenting effect of C99 on XE991 inhibition of KCNQ2/3 may reflect C99 modification of the XE991 binding site or of channel activation/pre-activation and its effects on inhibition by XE991.

### A potential role for KCNQ2/3 modulation in C99-linked pathology?

APP is thought to play a functional role in normal physiology at the nodes of Ranvier, modulating node formation and it may also promote nerve myelination [41]. APP is also required for appropriate migration of neuronal precursors into the cortical plate during development of the mammalian brain [22]. In addition, APP was previously found to modulate voltage gated sodium channels (Nav1.6) by enhancing their surface expression, and APP was found to co-localize with Nav1.6 in mouse cortical neurons [32].

Here, we found that proteins recognized by antibodies raised against either C99 or APP co-localize with KCNQ2, KCNQ3, and the nodal marker Ankyrin G. This positions APP, and an early cleavage product (C99) at a site at which they can interact with KCNQ2/3 *in vivo*. Accumulation of C99 at this site would therefore be predicted to inhibit KCNQ2/3 activity and could lead to aberrant neuronal signaling; we found that in oocytes, APP also co-immunoprecipitated with KCNQ2 but that its functional effects were weak compared to those of C99 (Fig. 5). This is of interest in AD because C99 has been identified as the earliest βAPP cleavage product and is implicated as an initiator of the neurodegeneration and changes in cognition in early AD, at least in the 3xTgAD mouse model [1]. C99 did not prevent the KCNQ2/3 current-augmenting effects of Kv channel openers retigabine and ICA27243, albeit these drugs could not rescue KCNQ2/3-C99 current to levels observed in the absence of C99. If C99 inhibition of KCNQ2/3 is in the future found to play a role in cognitive dysfunction in AD, it would be interesting to determine if KCNQ2/3 openers could ameliorate this effect.

APP, and its early catabolite, C99, are also found in other tissues outside the nervous system, with little being known of their function there [42]. Given the effects we observed, described herein, of C99 on Kv channels found in the brain and/or in other tissues, the possibility arises that C99 regulation of Kv channel activity occurs in various locations *in vivo*.

## Conclusion

We report that C99 is capable of modulating the activity of a variety of Kv channels in heterologous expression experiments. Furthermore, the KCNQ2/3 channel co-localized with APP/C99 in rat sciatic nerve nodes of Ranvier. As heterologous expression studies do not always recapitulate fully effects *in vivo*, future work is needed to reveal the possible role of C99 modulation of Kv channels in normal physiology, and to investigate the possibility of C99 regulation of Kv channels contributing to early cognitive defects in AD.
